# Roles of Regulatory RNAs for Antibiotic Resistance in Bacteria and Their Potential Value as Novel Drug Targets

**DOI:** 10.3389/fmicb.2017.00803

**Published:** 2017-05-05

**Authors:** Petra Dersch, Muna A. Khan, Sabrina Mühlen, Boris Görke

**Affiliations:** ^1^Department of Molecular Infection Biology, Helmholtz Centre for Infection ResearchBraunschweig, Germany; ^2^Department of Microbiology, Immunobiology and Genetics, Max F. Perutz Laboratories, University of ViennaVienna, Austria

**Keywords:** antibiotic resistance, non-coding RNA, small RNA, riboswitch, attenuation, antimicrobial chemotherapy, drug target, Hfq

## Abstract

The emergence of antibiotic resistance mechanisms among bacterial pathogens increases the demand for novel treatment strategies. Lately, the contribution of non-coding RNAs to antibiotic resistance and their potential value as drug targets became evident. RNA attenuator elements in mRNA leader regions couple expression of resistance genes to the presence of the cognate antibiotic. *Trans*-encoded small RNAs (sRNAs) modulate antibiotic tolerance by base-pairing with mRNAs encoding functions important for resistance such as metabolic enzymes, drug efflux pumps, or transport proteins. Bacteria respond with extensive changes of their sRNA repertoire to antibiotics. Each antibiotic generates a unique sRNA profile possibly causing downstream effects that may help to overcome the antibiotic challenge. In consequence, regulatory RNAs including sRNAs and their protein interaction partners such as Hfq may prove useful as targets for antimicrobial chemotherapy. Indeed, several compounds have been developed that kill bacteria by mimicking ligands for riboswitches controlling essential genes, demonstrating that regulatory RNA elements are druggable targets. Drugs acting on sRNAs are considered for combined therapies to treat infections. In this review, we address how regulatory RNAs respond to and establish resistance to antibiotics in bacteria. Approaches to target RNAs involved in intrinsic antibiotic resistance or virulence for chemotherapy will be discussed.

## Introduction

The emergence and spread of resistance to antibiotics represent a major threat for human health and urgently call for novel antimicrobial compounds and therapies. Traditionally, efforts to find novel treatment options have focussed on bacterial proteins as drug targets, whereas exploiting regulatory RNA elements was only considered of late. In bacteria, regulatory RNAs act at the post-transcriptional level to control bacterial physiology, development, and virulence (Oliva et al., 2015). Evidence is accumulating that regulatory RNAs are also important players for the bacterial response and resistance to antibiotics, making these molecules promising targets for antimicrobial chemotherapy.

Regulatory RNAs in bacteria comprise a heterogeneous group of molecules that act by various mechanisms to modulate cellular processes in response to cognate stimuli. These RNAs are often referred to as non-coding RNAs (ncRNAs) as they usually operate on their own without the need for being translated (Repoila and Darfeuille, 2009). Regulatory RNAs include two major classes, which are RNA attenuators and small RNAs (sRNAs) (Henkin, 2008). RNA attenuators are part of the mRNA that they regulate and therefore act *in cis*. Attenuators are sensory RNAs as they respond directly to environmental signals by toggling between alternative secondary structures either favoring or preventing expression of downstream genes (Naville and Gautheret, 2010; Mellin and Cossart, 2015). Classical attenuators monitor the ability of the ribosome to translate a short leader peptide. Another class of RNA attenuators comprises riboswitches, which respond to cognate small molecule ligands. The ligand binds to the riboswitch aptamer region and thereby alters the structure of an adjacent RNA element, i.e., the expression platform, dictating whether or not gene expression can occur. An additional major class of bacterial regulatory RNAs are sRNAs, which are expressed independently from their targets and distinguished as *cis*- or *trans*-encoded (Oliva et al., 2015): *cis*-encoded sRNAs, also called antisense RNAs, are transcribed in the opposite direction of their target genes and consequently they are fully complementary to their targets. Although there is an ongoing debate whether the often pervasive antisense transcription represents a meaningful response or simply reflects transcriptional noise (e.g., see Llorens-Rico et al., 2016), it became clear that antisense RNAs mediate a plethora of physiological effects through duplex formation with target transcripts (Georg and Hess, 2011). Finally, *trans*-encoded sRNAs regulate distantly encoded target RNAs by base-pairing through partial complementarity, but other mechanisms are also known (Storz et al., 2011). *Trans*-encoded sRNAs often rely on proteins, such as Hfq, ProQ, and CsrA for activity and function (Van Assche et al., 2015; Smirnov et al., 2016). In Gram-negative bacteria, Hfq accelerates sRNA/target RNA duplex formation, thereby modulating translation, decay, or transcription of the target RNA (Vogel and Luisi, 2011; Updegrove et al., 2016). As Hfq and CsrA are essential for the activity of numerous cognate sRNAs, their inhibition was shown to down-regulate sRNA networks controlling multiple virulence-relevant processes, which eventually can render bacteria not only non-infective but also more susceptible to antibiotics (Yamada et al., 2010; Oliva et al., 2015; Mühlen and Dersch, 2016). In the following chapters, we introduce recent advances in bacterial RNA research demonstrating the impact of various ncRNA classes on the resistance and tolerance to antimicrobials and discuss suitability of these riboregulators for antimicrobial chemotherapy.

## Implication of ncRNAs in Antibiotic Resistance and Tolerance

### Control of Antibiotic Resistance by RNA Attenuation – A Widespread Phenomenon

**FIGURE 1:**
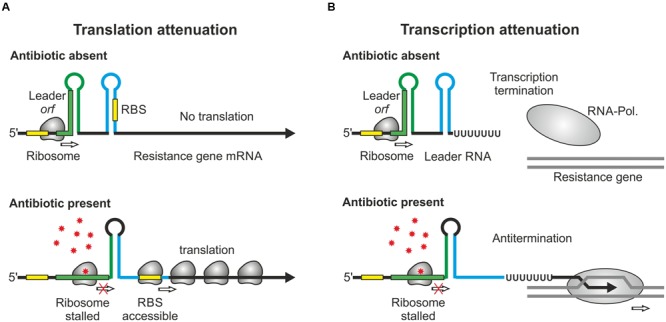
**Regulation of antibiotic resistance genes by RNA attenuation. (A)** Regulation by translational attenuation. The resistance gene *ermC* encodes a short *orf* in the leader region. When the leader *orf* is translated, the mRNA folds into a secondary structure, which represses translation of the resistance gene by sequestration of the RBS (top). Presence of the cognate antibiotic stalls the ribosome in the leader *orf*. This triggers formation of an alternative structure allowing ribosomes to access the RBS and to translate the resistance gene (bottom). **(B)** Regulation by transcriptional attenuation. In the case of *ermK* and similar attenuators (**Table 1**), translation of the leader *orf* causes the RNA-polymerase to terminate at an intrinsic terminator. Antibiotic-induced ribosome stalling in this *orf* favors formation of an antiterminator structure allowing RNA-polymerase to continue transcription beyond the terminator.

Over the past years, an ever-increasing number of studies reported mechanisms controlling antibiotic resistance genes at the post-transcriptional level. This type of regulation generates an immediate response, which is beneficial when antibiotic concentrations increase rapidly. RNA-based attenuation mechanisms are known to couple expression of resistance genes to presence of cognate antibiotics (**Table 1**). The classic example is provided by the *ermC* gene of *Staphylococcus aureus* and its variants, which confer resistance to macrolide antibiotics. They encode enzymes methylating a residue in 23S rRNA, which interferes with drug binding (Depardieu et al., 2007; Ramu et al., 2009). The leader region of the *ermC* mRNA encodes a short peptide (**Figure 1A**). Efficient translation of this *orf* triggers formation of an attenuator structure that sequesters the *ermC* ribosome binding site (RBS) shutting down translation. Binding of erythromycin causes the ribosome to stall, which allows formation of an alternative RNA structure in which the RBS is exposed, favoring translation (**Figure 1A**). Chloramphenicol as well as tetracycline resistance genes of *Bacteroides* are controlled by a similar mechanism (Schwarz et al., 2004; Wang et al., 2005). Importantly, translation attenuation is not simply the consequence of translation inhibition *per se* as each of the different attenuators exhibits a high specificity and responds to a different subset of antibiotics. Binding of the antibiotic by the translating ribosome alters the properties of the ribosomal peptidyl transferase center in a drug-specific manner, thereby inhibiting peptide bond-formation between specific combinations of amino acids that are present in the leader peptide (Marks et al., 2016).

**Table 1:** Regulatory RNAs contributing to antimicrobial resistance or susceptibility through known mechanisms.

Small RNA	Organism(s)	Resistance and/or inducer	Mechanism	Reference
**I. Attenuators and riboswitches**
*aac/aad*	Various species	Aminoglycosides	Riboswitch controlling translation of aminoglycoside acetyl- or adenyl-transferase genes	Jia et al., 2013
*bmrCD*	*Bacillus subtilis*	Antibiotics targeting the ribosome	Attenuator controlling transcription of *bmrCD* encoding an ABC transporter	Reilman et al., 2014
*cat*	Various species	Chloramphenicol	Attenuator controlling translation of chloramphenicol acetyltransferase genes	Schwarz et al., 2004
*cmlA*	Various species	Chloramphenicol	Attenuator controlling translation of chloramphenicol export genes	Schwarz et al., 2004
*ermC* (A, B)	Various species	MLS_B_	Attenuator controlling translation of ribosome methylase genes	Ramu et al., 2009
*ermK*	*Bacillus spec.*	MLS_B_	Attenuator controlling transcription of ribosome methylase genes	Kwak et al., 1991
*fexA*	*Staphylococcus lentus*	Chloramphenicol, florfenicol	Attenuator controlling translation of a chloramphenicol export gene	Schwarz et al., 2004
*lmo0919*	*Listeria monocytogenes*	Lincomycin	Attenuator controlling transcription of an ABC transporter gene	Dar et al., 2016
*mef*/*mel* (*msR*)	*Streptococcus*	Macrolides	Attenuator controlling transcription of an operon encoding a MFS efflux pump (Mef) and an ABC transporter (Mel)	Chancey et al., 2015
*tetM*	*Enterococcus faecalis*	Tetracycline	Attenuator controlling transcription of the ribosomal protection gene *tetM*	Su et al., 1992
*tetQ*	*Bacteroides*	Tetracycline	Attenuator controlling translation of the ribosomal protection gene *tetQ*	Wang et al., 2005
*vmlR*	*B. subtilis*	Lincomycin, virginiamycin M	Attenuator controlling transcription of *vmlR* encoding an ABC transporter	Ohki et al., 2005
**II. *Trans*-encoded sRNAs**			
DsrA	*E. coli*	Oxacillin, erythromycin, novobiocin	Overexpression provides resistance through upregulation of efflux pump MdtEF via RpoS	Nishino et al., 2011
GcvB	*E. coli*	D-cycloserine	GcvB provides resistance by repression of *cycA*, which is required for drug uptake	Pulvermacher et al., 2009
GlmY, GlmZ	*E. coli, Salmonella*	GlmS inhibitors (Bacilysin, Nva-FMDP)	Provide resistance via overproduction of GlmS	Khan et al., 2016
MicF	*E. coli, Salmonella*	Cephalosporins, norfloxacin	Deletion lowers and overexpression increases resistance through repression of *ompF*	Kim et al., 2015
MgrR	*E. coli*	Polymyxin B	MgrR mediates susceptibility by repressing synthesis of EptB, which modifies LPS	Moon and Gottesman, 2009
RybB	*E. coli*	Epigallocatechin gallate (EGCG)	EGCG activates expression of RybB, which down-regulates the biofilm regulator CsgD leading to inhibition of biofilm formation	Serra et al., 2016
RyhB	*E. coli*	Colicin Ia	RyhB mediates susceptibility by activation of synthesis of the colicin Ia receptor CirA	Salvail et al., 2013
SdsR (RyeB)	*E. coli*	Ampicillin	Ampicillin promotes mutations through repression of *mutS* by SdsR. Mutations may confer resistance	Gutierrez et al., 2013
SdsR (RyeB)	*E. coli, Salmonella*	Quinolones, novobiocin, crystal violet	Overexpression reduces resistance which is at least partially attributable to repression of *tolC* by SdsR	Kim et al., 2015; Parker and Gottesman, 2016
SroC	*Salmonella*	Polymyxin B	SroC contributes to resistance by downregulation of sRNA MgrR	Acuna et al., 2016
SprX (RsaOR)	*Staphylococcus aureus*	Glycopeptides	Overexpression reduces and deletion increases resistance. SprX acts by repression of *spoVG*.	Eyraud et al., 2014
3′ETS*^leuZ^*	*E. coli*	Colicin Ia	Contributes to resistance by lowering RyhB levels	Lalaouna et al., 2015

*MLS_*B*_, Macrolides, lincosamides, streptogramin B.*

A variation of this attenuation mechanism is known to control transcription elongation rather than translation and is used to regulate expression of the macrolide resistance genes encoded by *ermK* and the *mef*-*mel (msr)* operon in *Bacillus* and *Streptococcus* species (Kwak et al., 1991; Chancey et al., 2015). In the absence of macrolides, transcription stops at a formed terminator structure present in the leader regions of the resistance genes (**Figure 1B**). Antibiotic-induced ribosome stalling within the short leader *orfs* favors formation of anti-terminator structures allowing RNA-polymerase to continue transcription (**Figure 1B**). A similar mechanism regulates *vmlR* and *bmrCD*, which encode ABC transporters conferring antibiotic resistance in *Bacillus subtilis* (Ohki et al., 2005; Reilman et al., 2014) (**Table 1**). In this case, dedicated leader peptides are not detectable. Distinct to previous systems, expression of *bmrCD* is regulated via a transcriptional attenuator located upstream in the *bmrB* gene. Translation of *bmrB* is essential for *bmrCD* regulation suggesting that it takes over the role of a leader peptide (Reilman et al., 2014).

Recently, an RNA element was claimed to interact directly with aminoglycoside antibiotics to achieve regulated expression of downstream encoded aminoglycoside acetyl- or adenyl-transferases (Jia et al., 2013). The interaction was proposed to trigger a conformational change in the leader RNA, thereby unmasking the RBS, which is sequestered in a stem-loop in the absence of a ligand. However, this mechanism bearing the characteristics of a genuine riboswitch is still a matter of debate (He et al., 2013; Roth and Breaker, 2013).

Until recently, regulation of antibiotic resistance gene expression by transcriptional attenuation was considered a rare mechanism as only few cases were known. Most attenuator and riboswitch elements were discovered by studying individual genes or by comparative genomics searching for conserved elements in leader sequences. However, a new experimental approach termed Term-seq, developed for a genome-wide search of transcriptional attenuators responding to a metabolite of choice, revealed many additional attenuators and riboswitches responding to antibiotics (Dar et al., 2016). This platform combines genome-wide mapping of transcriptional start sites with a protocol mapping all RNA 3′ termini to identify transcriptional termination events in RNA leaders. One of the novel attenuators detected in *Listeria monocytogenes*, was analyzed in detail and shown to regulate expression of an ABC-transporter in response to lincomycin (**Table 1**). Deletion analysis demonstrated that this transporter is important for lincomycin resistance. Thus, Term-seq not only identifies novel antibiotic-responsive RNA elements but also novel resistance genes controlled by these riboregulators. Applying Term-seq to the human oral microbiome revealed that this type of regulation is widespread and very common in Gram-positive bacteria (Dar et al., 2016).

### Regulatory Networks Controlling Antibiotic Resistance Include Small RNAs

**FIGURE 2:**
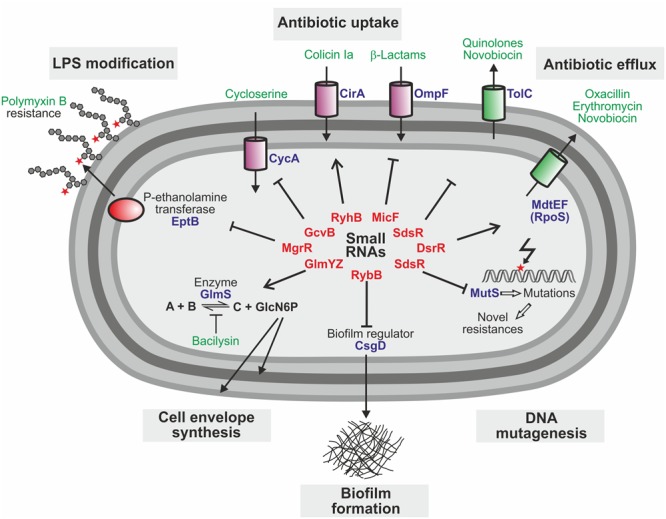
***Trans*-encoded sRNAs with impact on antibiotic resistance and susceptibility in *E. coli*.** Cartoon summarizing the known roles of sRNAs and their targets for resistance to antimicrobials in *E. coli*. Small RNAs are typed in red, target proteins in blue, and antibiotics in green.

There is accumulating evidence that *trans*-encoded sRNAs are also key players in regulatory circuits controlling antibiotic resistance (**Table 1**). These circuits govern various processes (**Figure 2**), including functions required for antibiotic uptake (Pulvermacher et al., 2009; Salvail et al., 2013; Kim et al., 2015; Lalaouna et al., 2015), modifications of the cell envelope shielding against antimicrobials (Moon and Gottesman, 2009; Acuna et al., 2016), drug efflux pumps expelling antibiotics (Nishino et al., 2011; Parker and Gottesman, 2016), metabolic enzymes conferring resistance (Khan et al., 2016), production of biofilms protecting from antibiotics (Serra et al., 2016) and DNA mutagenesis mechanisms facilitating evolution of novel resistances (Gutierrez et al., 2013). The different *trans*-encoding sRNAs may regulate expression of resistance genes either directly by base-pairing or indirectly as members of regulatory cascades coordinating the response to antibiotics.

The sRNAs MicF, GcvB, and RyhB modulate antibiotic resistance in *E. coli* by regulation of genes required for antibiotic uptake (**Figure 2** and **Table 1**). MicF represses translation of the OmpF porin, a major antibiotics uptake pathway (Nikaido, 1989). Consequently, deletion of *micF* increases, whereas overexpression decreases susceptibility to antibiotics such as cephalosporin and norfloxacin (Kim et al., 2015). Similarly, the absence of the sRNA GcvB increases susceptibility to D-cycloserine (Pulvermacher et al., 2009). GcvB base-pairs with and represses the mRNA of the serine transporter CycA, which also transports D-cycloserine. Finally, the iron-responsive sRNA RyhB sensitizes *E. coli* to colicin Ia (Salvail et al., 2013). Colicins are toxins that are produced by some *E. coli* strains to suppress competitors by depolarization of the cytoplasmic membrane. Susceptibility to colicin Ia strongly increases upon iron starvation as these conditions upregulate the iron-siderophore receptor CirA, which translocates colicin Ia into the periplasm. Activation of CirA synthesis requires RyhB, which accumulates under iron depletion conditions and stimulates *cirA* translation. Accordingly, *ryhB* mutants are impaired in colicin Ia uptake, providing resistance (Salvail et al., 2013).

**FIGURE 3:**
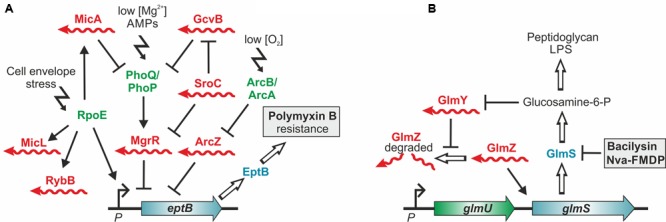
**Control of antibiotic resistance by *trans*-encoded sRNAs. (A)** Control of polymyxin resistance in *E. coli*. Enzyme EptB provides resistance to polymyxin B by modification of LPS with phosphoethanolamine. Translation of *eptB* mRNA is inhibited by sRNA MgrR, which is itself repressed by base pairing with the sponge sRNA SroC. Consequently, loss of MgrR increases and loss of SroC decreases resistance to polymyxin B. In addition, *eptB* is repressed by sRNA ArcZ (Moon et al., 2013), whose levels are controlled by the aerobic/anaerobic-sensing ArcA–ArcB two-component system (Mandin and Gottesman, 2010). Counterintuitively, deletion of Hfq, which is required for activity of these sRNAs increases susceptibility to polymyxin B. This might be explained by a defective cell envelope stress response executed by RpoE. RpoE not only activates transcription of *eptB* but also of further Hfq-dependent sRNAs, which control LPS biogenesis and modification. Complexity is further increased by the fact that *mgrR* transcription is activated by the two-component system PhoQ/PhoP, which is repressed by sRNAs MicA and GcvB. **(B)** sRNA-mediated resistance to antibiotics targeting the cell wall biosynthesis enzyme GlmS. In *Enterobacteriaceae* small RNAs GlmY and GlmZ feedback-regulate GlmS synthesis to achieve homeostasis of the essential metabolite GlcN6P. Inhibition of GlmS by bacilysin and other antibiotics depletes GlcN6P, which is sensed by sRNA GlmY triggering its accumulation. By a mimicry mechanism GlmY counteracts degradation of the homologous sRNA GlmZ, which in turn selectively activates translation of *glmS* encoded within the *glmUS* operon. As a result, higher GlmS levels are produced compensating for its inhibition.

The sRNA MgrR controls modification of the cell envelope and thereby mediates susceptibility of *E. coli* to the cationic antimicrobial peptide polymyxin B (Moon and Gottesman, 2009). MgrR represses translation of the *eptB* mRNA, which encodes a protein that modifies lipopolysaccharides (LPS) with phosphoethanolamine (**Figures 2, 3A**). Absence of MgrR causes higher EptB levels leading to extensive LPS modifications, which reduce the net anion charge of LPS and prevent polymyxin B binding. Another class of sRNAs emerged, which also affects antibiotic resistance by acting as sponges for other sRNAs (Miyakoshi et al., 2015; Bossi and Figueroa-Bossi, 2016) (**Table 1**). One of these is the sRNA SroC which binds and sequesters the sRNA MgrR (Acuna et al., 2016) (**Figure 3A**). Consequently, a *sroC* deletion increases free MgrR levels and enhances susceptibility to polymyxin B (Acuna et al., 2016). Similarly, an excised spacer of a tRNA precursor named 3′ETS*^leuZ^* base-pairs with several sRNAs in *E. coli*, including MicF and RyhB, to adsorb transcriptional noise when these sRNAs are repressed (Lalaouna et al., 2015). Accordingly, higher levels of RyhB are obtained upon mutation of 3′ETS*^leuZ^* rendering the bacteria more susceptible to colicin Ia.

Several sRNAs were shown to regulate genes for drug efflux pumps, which expel antibiotics from the cell (**Figure 2**). In *E. coli* and *Salmonella*, SdsR binds and represses the *tolC* mRNA, which encodes the common component of efflux pumps including the broad spectrum AcrAB system, which exports lipophilic antibiotics (Kim et al., 2015; Fröhlich et al., 2016; Parker and Gottesman, 2016). Consequently, overexpression of SdsR reduces resistance to novobiocin and several quinolone antibiotics (Kim et al., 2015; Parker and Gottesman, 2016). Two additional sRNAs, DsrA in *E. coli* and NrrF in *Neisseria gonorrhoeae*, were found to regulate multi-drug efflux pump genes and for DsrA an effect on antibiotic resistance was shown (Nishino et al., 2011; Jackson et al., 2013) (**Table 1**).

The overall importance and impact of sRNAs on the regulation of antibiotic resistance became even more evident by a recent work in which the influence of sRNAs was assessed in a more systematic manner (Kim et al., 2015). A library of *E. coli* strains overproducing or lacking individual sRNAs was screened for altered susceptibility to various clinically relevant antibiotics. Interestingly, overexpression of 17 out of 26 tested sRNAs affected resistance or susceptibility to antibiotics. Most of these sRNAs generated identical effects in *Salmonella* suggesting conservation of the underlying mechanisms, but only a few generated opposite phenotypes in equivalent sRNA knock-out strains (Kim et al., 2015). This cannot be easily explained, but one obstacle in the analysis is that overexpression of a particular Hfq-binding RNA can affect antibiotic resistance indirectly by sequestration of the RNA chaperone Hfq, thereby outcompeting other Hfq-binding RNAs (Papenfort et al., 2009; Moon and Gottesman, 2011).

In summary, extensive work in recent years indicates that *trans*-encoded sRNAs are important elements in controlling antibiotic resistance genes in *E. coli* and *Salmonella*, where these regulators were most thoroughly investigated (**Figure 2**). A recent report of a sRNA mediating susceptibility of *S. aureus* to glycopeptide antibiotics (Eyraud et al., 2014) (**Table 1**), which are invaluable drugs for treatment of methicillin-resistant staphylococcal infections, suggests that this might also hold true for other pathogenic bacteria.

## Are Small Rnas Crucial Elements For The Bacterial Response To Antibiotics?

Implication of sRNAs in antibiotic resistance control suggests that not only attenuator elements but also *trans*-encoded sRNAs could be controlled in response to antibiotics. In fact, the application of omics technologies revealed that sub-MIC concentrations of antibiotics provoke compound-specific effects on the bacterial transcriptome and proteome. Importantly, these changes contribute to antibiotic tolerance, helping the bacteria to overcome growth inhibition (Wecke and Mascher, 2011; Laureti et al., 2013; Pulido et al., 2016). Recent analyses indicated that the changes of the transcriptional profile in response to antibiotics also include sRNAs.

Initial studies with different bacterial species reported that the levels of individual sRNAs are altered after antimicrobial treatment (Oh et al., 2000; Chen et al., 2011; Perez-Martinez and Haas, 2011; Yu and Schneiders, 2012). For instance, a study in *Salmonella* Typhimurium identified four sRNAs accumulating upon tigecycline treatment (Yu and Schneiders, 2012). Notably, deletion of one, SroA, increased susceptibility to tigecycline and ectopic expression rescued resistance. Similarly, in *Clostridia* the antibiotic clindamycin induces an sRNA, which is encoded immediately upstream of an ABC transporter, whose homolog confers clindamycin resistance in *Staphylococcus* species (Chen et al., 2011), indicating that antibiotic-responsive sRNAs might be part of a bacterial defense strategy.

Now, in-depth transcriptome analyses revealed that antibiotics elicit significant changes in the bacterial sRNA repertoire that are much more extensive than previously envisioned (Howden et al., 2013; Stubben et al., 2014; Jeeves et al., 2015; Molina-Santiago et al., 2015). More precisely, upregulation of certain antisense RNAs was detected in methicillin-resistant *Staphylococcus aureus* as well as in *Mycobacterium tuberculosis* upon exposure to antibiotics frequently used to treat corresponding infections and in a multi-resistant *Pseudomonas putida* strain 140 candidate sRNAs were detected, which responded to at least one of multiple tested antibiotics (Howden et al., 2013; Jeeves et al., 2015; Molina-Santiago et al., 2015). Of note, each antibiotic generated a unique sRNA expression profile. Some antibiotics impacted the expression of dozens of sRNAs, whereas others affected only a few (Molina-Santiago et al., 2015). All these observations are also in favor of a bacterial program in which sRNAs orchestrate responses to antibiotics. Further work is required to discriminate direct from indirect effects and to determine whether provoked sRNA profile changes contribute to drug tolerance.

**FIGURE 4:**
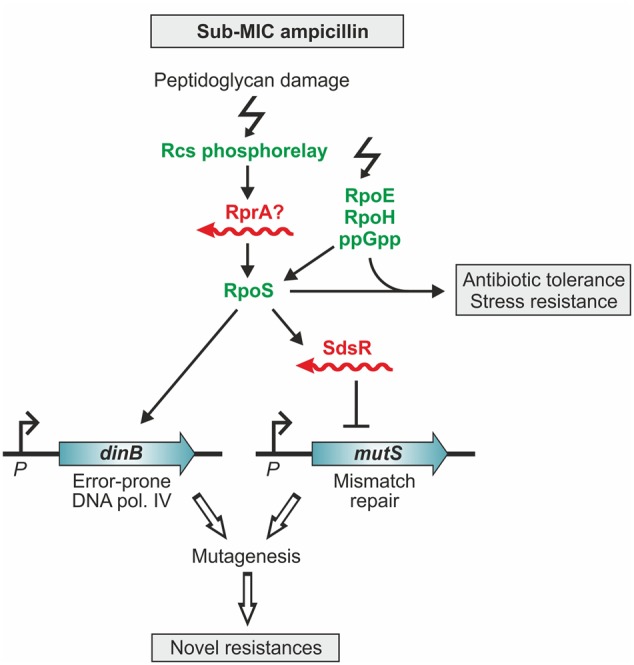
**The response of *E. coli* to sublethal concentrations of ampicillin involves small RNAs.** Sub-MIC concentrations of ampicillin induce the stress regulons controlled by RpoS, RpoE, RpoH, and the alarmone ppGpp (Mathieu et al., 2016). The resulting hormetic response renders cells resistant to higher ampicillin concentrations and other stresses. Induction of the RpoS-regulated general stress response occurs via accumulation of ppGpp and the Rcs phospho-relay system. Rcs senses peptidoglycan damage caused by ampicillin and activates RpoS via an Hfq-dependent sRNA, presumably RprA (Majdalani et al., 2002; Mathieu et al., 2016). Induction of the RpoS regulon also increases the level of the error-prone polymerase IV, which generates base-substitutions in the DNA (Gutierrez et al., 2013). The introduced mutations become fixed because the levels of the mismatch repair protein MutS decrease upon ampicillin treatment. RpoS represses *mutS* indirectly by activating expression of sRNA SdsR, which downregulates *mutS* by base pairing. This mechanism leads to increased mutagenesis, which can generate mutations conferring antibiotic resistance (Gutierrez et al., 2013).

Antibiotics at sub-MIC concentrations not only trigger adaptive responses that enable bacteria to survive successive exposures to higher antibiotic concentrations and other lethal stresses (Mathieu et al., 2016; Mitosch et al., 2017), but even have effects beyond: They increase mutation rates promoting emergence of novel antibiotic resistances and also stimulate the spread of resistance genes by horizontal transfer (Baharoglu and Mazel, 2011; Gullberg et al., 2011; Laureti et al., 2013). In *E. coli*, sub-MICs of antibiotics activate the master regulator of the general stress response, RpoS, which holds a key role in the latter processes (Gutierrez et al., 2013; Mathieu et al., 2016). The *rpoS* mRNA represents a hub for post-transcriptional regulation as it is positively and negatively controlled by base-pairing with multiple sRNAs including RprA (Zhang et al., 1998; Sedlyarova et al., 2016). One of these sRNAs apparently contributes to the induction of RpoS in response to ampicillin (**Figure 4**) (Mathieu et al., 2016). The cell wall damages caused by β-lactam antibiotics are sensed by the Rcs phosphorelay signal transduction system, which triggers activation of RpoS (**Figure 4**). However, upregulation of RpoS is indirect and occurs through an Hfq-dependent sRNA. The likely sRNA candidate is RprA because its expression is positively controlled by the Rcs system. Induction of RpoS not only activates genes counteracting stress, but also upregulates the error-prone DNA polymerase IV (PolIV), which incorporates spontaneous mutations (**Figure 4**) (Gutierrez et al., 2013; Mathieu et al., 2016). Moreover, RpoS activates expression of sRNA SdsR, which down-regulates the DNA mismatch repair protein MutS, thereby favoring fixation of the mutations introduced by PolIV (Gutierrez et al., 2013). This mechanism increases genetic diversity, which could lead to mutations conferring antibiotic resistance (**Figure 4**). According to a study in *Salmonella*, RpoS and the sRNA RprA are also important for plasmid conjugation and could potentially have an impact on horizontal transfer of antibiotic resistance genes (Papenfort et al., 2015).

## Regulatory and Sensory RNAs As Valuable Drug Targets

### Targeting Riboswitch Elements for Antimicrobial Chemotherapy

Previous attempts to target specific RNA structures (e.g., ribosomal RNAs) have shown that this is a valuable task, as it has led to the identification of many natural compounds and derivatives thereof that are now used in antimicrobial therapies (Hermann and Westhof, 1998; Hong et al., 2014). In general, riboswitches display a high affinity and specificity for their endogenous ligands and the majority controls the expression of virulence-relevant/essential metabolic genes (Blount and Breaker, 2006; Lünse et al., 2014). The idea to target riboswitches is reinforced by discoveries, showing that well-known antimicrobial compounds (e.g., thiamine analog pyrithiamine; lysine analog DL-4-oxalysine), whose mode of action remained unknown for decades, act through riboswitches (Sudarsan et al., 2005; Blount et al., 2007).

Riboswitch classes are known that interact with ions or certain metabolites. Meanwhile, a number of antibacterial small molecule inhibitors was identified that mimic the natural ligands and influence riboswitch-controlled functions by binding selectively to the corresponding riboswitch. Some of the most potent compounds silence essential genes of bacteria, which respond to lysine, glucosamine-6-phosphate (GlcN6P), purine, cyclic-di-GMP, flavin mononucleotide (FMN) and thiamine pyrophosphate (Lünse et al., 2014; Matzner and Mayer, 2015; Schüller et al., 2016). Approaches using synthetic mimics of the natural ligands further demonstrated that riboswitches are druggable by synthetic chemistry (Howe et al., 2015). Considering the wide distribution of some riboswitches (e.g., FMN-riboswitches), the interacting compounds can be used as broad-spectrum anti-infective, whereas those with a more species-specific target will be more selective (Barrick and Breaker, 2007).

Several of the identified riboswitch-targeting compounds inhibit bacterial growth and effectively kill bacteria under *in vitro* growth conditions, demonstrating their potency as therapeutic agents (Blount et al., 2007; Kim et al., 2009). A few have proven to reduce pathogenicity in animal infection models. Among them is ribocil, which mimics the ligand FMN of the riboflavin riboswitch and prevents riboflavin biosynthesis (Howe et al., 2015). In an *E. coli* septicemia mouse model ribocil reduced the bacterial burden by 2–3 orders of magnitude, demonstrating its efficacy to inhibit riboflavin biosynthesis. Another example is 2,5,6-triaminopyrimidine-4-one (PC1), a guanine analog inhibiting expression of riboswitch-controlled guanosine monophosphate synthesis in *S. aureus*. Administration of PC1 reduced the number of *S. aureus* in the mammary glands of infected mice and in the milk of cows (Mulhbacher et al., 2010; Ster et al., 2013).

Despite these promising results, attention must be paid to the emergence of resistances to riboswitch analogs by mutations in the aptamer region (Sudarsan et al., 2005). Moreover, undesired off-targets effects need to be considered due to possible interactions of metabolite analogs with other enzymes utilizing these ligands as cofactors, as seen with the riboflavin analog roseoflavin (Mansjö and Johansson, 2011). Another obstacle is that most ligands are highly charged. They cannot passively pass the cell envelope and need to be optimized to allow their path into clinical settings. Nonetheless, work on riboswitches provided the proof that regulatory RNA elements are indeed druggable and suitable targets for antimicrobial chemotherapy.

### Targeting *Trans*-encoded sRNAs and Their Protein Interaction Partners

The roles of *trans*-encoded sRNAs for antimicrobial resistance are just emerging and strategies to exploit them for chemotherapy are still in their infancies. Targeting these regulators will not lead to bacterial death directly, but may provide fitness reduction and the possibility to amplify efficacy of existing antibiotics in combined therapy. Drugs interfering directly with *trans*-encoded sRNA function *in vivo* are currently unknown, but might be feasible as for riboswitches. Alternatively, compounds modulating sRNA levels could be useful to boost antibiotic activity, as suggested by two recent studies. The first example involves the two homologous sRNAs GlmY and GlmZ, which feed-back regulate synthesis of enzyme GlmS in enteric bacteria (Göpel et al., 2014) (**Figures 2, 3B**). GlmS initiates cell envelope synthesis by generating the key metabolite GlcN6P. The sRNAs accumulate upon depletion of this metabolite and in turn stimulate *glmS* translation to replenish the GlcN6P pool. This mechanism also provides protection against antibiotics such as bacilysin, which act by inhibition of GlmS (Khan et al., 2016). The resulting drop of GlcN6P induces the sRNAs, which in turn trigger *glmS* overexpression thereby overcoming growth inhibition by the antibiotic (**Figure 3B**). Consequently, the bactericidal potency of GlmS inhibitors can be increased by co-application of a non-metabolizable GlcN6P analog, which suppresses accumulation of GlmY/GlmZ (Khan et al., 2016). The second example is provided by the *E. coli* sRNA RybB, targeting the mRNA encoding the crucial biofilm regulator CsgD (**Figure 2**). Epigallocatechin gallate (EGCG), a polyphenol present in green tea, was found to activate *rybB* expression, which abolishes biofilm formation and affects biofilm-mediated resistance against antibiotics and host defenses (Serra et al., 2016), making EGCG a promising adjuvant that increases antibiotic susceptibility in combined chemotherapy.

*Trans*-encoded sRNAs frequently rely on RNA chaperones and RNA-binding proteins such as Hfq, ProQ or CsrA for function (Van Assche et al., 2015; Smirnov et al., 2016). As these proteins are required for virulence of many bacteria (Vogel and Luisi, 2011; Vakulskas et al., 2015; Heroven et al., 2016), they represent excellent targets for anti-infective strategies (Mühlen and Dersch, 2016). Importantly, mutation of Hfq not only attenuates virulence but also increases susceptibility to antibiotics (Yamada et al., 2010), which could also reflect the roles of Hfq-dependent *trans*-encoded sRNAs in this process (**Table 1**). However, the effect of Hfq inactivation on individual resistance genes is difficult to predict, because they are often controlled by extensive regulatory networks involving multiple sRNAs (**Figure 3A**). For instance, *eptB*, which provides resistance to polymyxin B, is repressed by the Hfq-dependent sRNA MgrR (**Figure 3A**). However, deletion of Hfq counterintuitively increases susceptibility of uropathogenic *E. coli* to polymyxin B (Kulesus et al., 2008). The reason for this opposing effect is unclear, but might be attributable to the influence of Hfq-dependent sRNAs on the RpoE-dependent cell envelope stress response and thus envelope integrity, or the control of the MgrR sRNA by the two-component system PhoP/PhoQ, which is also regulated by Hfq-dependent sRNAs (**Figure 3A**) (Gogol et al., 2011; Moon et al., 2013; Guo et al., 2014). One of these sRNAs is GcvB (Coornaert et al., 2013), which is repressed by base-pairing with the sponge sRNA SroC, similar to MgrR (Miyakoshi et al., 2015). However, whether downregulation of GcvB by SroC affects *eptB* expression remains to be clarified. This example illustrates that thorough knowledge of the complex regulatory network governing a resistance gene is a prerequisite to avoid unpredictable effects of this class of inhibitors.

Meanwhile, a first inhibitor of Hfq-sRNA interactions has been identified (El-Mowafi et al., 2014). Using an intein-based technology, a library of cyclic peptides was screened for inhibition of sRNA-target RNA interaction in *E. coli*. A peptide named RI20 inhibits Hfq function *in vivo*, even when added exogenously. RI20 is predicted to bind to the proximal site of Hfq, which is required for interaction with most sRNAs (Updegrove et al., 2016), and therefore likely inhibits Hfq-sRNA interactions globally. This broad specificity would not only suppress virulence functions but would also make cells more amenable to antibiotic chemotherapy. Indeed, RI20 increases susceptibility of *E. coli* to antibiotics, pheno-copying an *hfq* mutant. These results demonstrate that Hfq is a druggable target and provide an experimental setup for identification of more potent inhibitors (El-Mowafi et al., 2014).

CsrA is a global RNA-binding protein that modulates mRNA expression by interfering with translation initiation. Its activity is regulated by dedicated sRNAs, which sequester and thereby counteract CsrA. Importantly, CsrA coordinates the expression of virulence factors in many pathogens (Vakulskas et al., 2015; Heroven et al., 2016). Deficiency of CsrA impairs colonization of the host leading to attenuated virulence. Recently, a two-step strategy was applied to find compounds inhibiting interaction of *Yersinia pseudotuberculosis* CsrA with RNA (Maurer et al., 2016). First, a surface plasmon resonance assay was used to identify compounds binding to CsrA. The identified molecules were subsequently subjected to fluorescence polarization-based competition assays to test for inhibition of CsrA-RNA interaction, resulting in identification of a myxobacterial metabolite as most potent inhibitor. In a parallel approach, a rational ligand-based strategy was applied to identify a tri-nucleotide GGA RNA, which inhibits CsrA-RNA interaction by mimicking the CsrA binding motif. The identified compounds are promising starting points for the development of high-affinity inhibitors, which could later be applied to *in vivo* models (Maurer et al., 2016).

## Conclusion and Perspectives

To avoid costs of energy and fitness, bacteria frequently express antibiotic resistance genes in a regulated manner and growing evidence suggests that ncRNAs play pivotal roles in the control of this process. Riboregulation is fast as it allows to target preexisting RNA and is easier to evolve as compared to protein-based regulation (Updegrove et al., 2015) – features that are beneficial for the evolution of antibiotic resistance. Resistance genes that encode antibiotic efflux transporters or enzymes modifying the ribosome are often controlled by attenuation mechanisms operating in their RNA leader regions (**Table 1**). These genes operate independently of other factors, can be easily mobilized and transferred to other species (Chancey et al., 2012), and their inheritance allows instant control of the resistance gene in the recipient. Pioneering research in *E. coli* and *Salmonella* has revealed that *trans*-encoded sRNAs contribute extensively to intrinsic antibiotic resistance and susceptibility. sRNAs participate in complex regulatory circuits controlling antibiotic transporters or efflux pumps or other functions relevant for antibiotic action such as cell envelope synthesis and modification (**Figure 2** and **Table 1**). It remains to be seen whether this also applies to other bacterial species.

Bacteria respond with specific changes of the transcriptome to cope with antibiotic stress and recent observations suggest that ncRNAs are involved. This definitely applies to transcriptional attenuation mechanisms, which are much more frequent and widespread than previously thought (Dar et al., 2016). Whether antibiotic-induced changes in sRNA levels are essential to orchestrate cellular defense strategies or whether they simply reflect an indirect and unspecific consequence of the antibiotic action needs to be shown.

Targeting regulatory RNAs provides the opportunity to increase efficacy of existing antibiotics by silencing of corresponding resistance genes in combined therapy. Research on riboswitches provided the proof that bacterial regulatory RNAs are druggable *in vivo* suggesting that RNAs controlling antibiotic resistance can be targeted in a similar way. Recent progress in targeting microRNAs (Childs-Disney and Disney, 2016), the eukaryotic counterparts of sRNAs, is in favor of this idea. In addition, antibiotic efficacy could be improved by manipulating the levels of sRNAs involved in resistance. This can be accomplished either by targeting upstream regulators of individual sRNAs or more globally by inhibition of sRNA-binding proteins such as Hfq, which is required for sRNA function and stability. Promising inhibitors of CsrA and Hfq activity have been identified and now await further optimization and evaluation with appropriate infection models.

## Author Contributions

PD and BG formulated the outline. All authors contributed to writing and approved the final manuscript for publication.

## Conflict of Interest Statement

The authors declare that the research was conducted in the absence of any commercial or financial relationships that could be construed as a potential conflict of interest.
